# The Comparative Immunological Characteristics of SARS-CoV, MERS-CoV, and SARS-CoV-2 Coronavirus Infections

**DOI:** 10.3389/fimmu.2020.02033

**Published:** 2020-08-14

**Authors:** Yun-yu Zhang, Bi-ru Li, Bo-tao Ning

**Affiliations:** Department of Paediatric Intensive Care Unit, Shanghai Children's Medical Center, School of Medicine, Shanghai Jiao Tong University, Shanghai, China

**Keywords:** novel coronavirus, SARS-CoV-2, SARS-CoV, MERS-CoV, immunology

## Abstract

Immune dysfunction and aberrant cytokine storms often lead to rapid exacerbation of the disease during late infection stages in SARS-CoV and MERS-CoV patients. However, the underlying immunopathology mechanisms are not fully understood, and there has been little progress in research regarding the development of vaccines, anti-viral drugs, and immunotherapy. The newly discovered SARS-CoV-2 (2019-nCoV) is responsible for the third coronavirus pandemic in the human population, and this virus exhibits enhanced pathogenicity and transmissibility. SARS-CoV-2 is highly genetically homologous to SARS-CoV, and infection may result in a similar clinical disease (COVID-19). In this review, we provide detailed knowledge of the pathogenesis and immunological characteristics of SARS and MERS, and we present recent findings regarding the clinical features and potential immunopathogenesis of COVID-19. Host immunological characteristics of these three infections are summarised and compared. We aim to provide insights and scientific evidence regarding the pathogenesis of COVID-19 and therapeutic strategies targeting this disease.

## Introduction

It was not until the pandemic outbreak of severe acute respiratory syndrome (SARS) in 2003 that people came to realise that devastating zoonotic diseases could be caused by coronavirus strains, and this realisation raised concerns regarding the potential health threats of these viral strains to the human population. Thus far, six strains of human CoVs have been identified, and three of these strains are highly pathogenic (SARS-CoV, MERS-CoV, and SARS-CoV-2 [2019-nCoV]) and are capable of inducing lethal pneumonia and systematic symptoms in humans. These viruses are very similar in their genome composition, their routes of infection transmission, and their host clinical manifestations. The recently discovered SARS-CoV-2 (also designated as SARS-CoV-2 by the International Committee on Taxonomy of Viruses [ICTV]) is believed to share many similarities with severe acute respiratory syndrome (SARS). Amid rising debates and controversies, the World Health Organisation (WHO) clearly defined this specific infectious disease as “COVID-19” (Corona virus disease-19) (1). COVID-19 is currently creating an unprecedented health challenge for all nations and nationalities. More than 10 million cases have been reported in over 200 countries (2), and the case fatality rate varies from 1.4 to 6.9% (Table 1). For critically ill patients, the case fatality rate can be as high as 49% (14). Successive transnational and community outbreaks are still occurring worldwide, and no effective therapeutic measures have currently been proposed.

**Table 1:** Epidemiology, virology, demography, and clinical characteristics of SARS-CoV, MERS-CoV, and SARS-CoV-2.

	**SARS-CoV (3–6)**	**MERS-CoV (4, 6–8)**	**SARS-CoV-2 (7, 9–13)**
**1. Epidemiology**			
Number of countries affected (*n*)	29	27	216
Confirmed cases (*n*)	8,096	2,494	>10 million
Basic reproduction number	0.3–4.1	<1	2.2–2.6
Mean Incubation period (range)	4.6 (2–14)	5.2 (2–13)	5.1 (2.2–14)
Severe cases ratio (%)	34%	63.4%	15.7%
Crude case fatality rate (%)	9.6%	40%	1.4-6.9%
Case fatality rate in patients with comorbidities (%)	46%	60%	11%
**2. Virology**			
Proposed intermediate host	Palm civets	Camels	Malayan pangolins
Cellular entry receptor	ACE2	DPP4	ACE2
Sequence identity to SARS-CoV-2	79.5%	50%	/
Potential viral-host mechanism	Interferon antagonism, abortive infection (macrophage, dendritic cell, lymphocyte), T cell functional exhaustion, IMM infiltration	Interferon antagonism (repressive histone modification), antibody dependent enhancement (ADE), MHC gene down-regulation, T cell functional exhaustion	Interferon antagonism, abortive infection, antibody dependent enhancement (ADE), altered monocyte signature profile, macrophage polarisation? T cell functional exhaustion?
**3. Demography and clinical signs**			
Median age of patients (range)	39.9 (1–91)	47 (1–94)	56(<0–>80)
Sex ratio (male:female)	43%:57%	64.5%:34.5%	58.1%:41.9%
Typical clinical presentation	Acute pneumonia in the elderly and patients with comorbidities; flu-like symptoms or asymptomatic infection in immunocompetent patients	Progressive acute lethal pneumonia in all infected patients	Acute pneumonia in the elderly and patients with comorbidities; flu-like symptoms, asymptomatic infection in immunocompetent patients and children
Extra pulmonary injuries/symptoms	Diarrhoea	Acute renal failure, diarrhoea	Headache, nausea, vomiting, diarrhoea
Comorbidities ratio	10–30%	76%	48%
**4. Clinical course and disease progression**			
Medium days of peak viral load	Day 10 after symptom onset	≥Day14 after symptom onset	At symptom onset
Onset time of neutralising antibodies	>Day14	>Day12	>Day 10
Median days from onset of symptoms to hospital admission	2 days	4 days	4 days
Median days from onset of symptoms to ICU admission (developed ARDS)	6.5 days	5 days	10 days
Median days from onset of symptoms to death	23.7 days	11.5 days	18.5 days
Risk factors related to disease progression or mortality	Age, comorbidities (diabetes, HBV infection), LDH level, high neutrophils, low CD4 and CD8 lymphocytes counts	Age, male, comorbidities (diabetes, chronic renal disease), immunocompromised state	Age, comorbidities (COPD, heart disease), elevated d-dimers, inflammatory indicators, increased neutrophil/lymphocytes ratio

SARS-CoV-2 possesses a typical “corona”-like structure when viewed under an electron microscope, and this virus shares a similar host cellular entry mechanism with SARS-CoV that involves binding to the human angiotensin-converting enzyme 2 (ACE2) receptor through its surface protein receptor binding domain (RBD) (15, 16). In contrast, MERS-CoV achieves host cellular entry via binding to the DPP4 receptor (17). According to the results of genomic analyses, SARS-CoV-2 shares a 79.5% sequence identity to SARS, while only sharing a 50% sequence identity with MERS-CoV (18) (Table 1). In an attempt to elucidate the possible evolutionary origin of COVID-19, it was determined that this virus was 96% genetically identical to a bat-derived coronavirus discovered in 2013 (19). At the amino acid level, 380 substitution sites were identified when COVID-19 was compared to SARS-CoV (15). Based on this, it is reasonable to hypothesise the existence of potential novel viral protein functions and undefined pathogenesis. For example, variations in the spike structural protein (S protein) and nucleocapsid protein (N protein) may be responsible for the higher transmissibility and lower pathogenicity of SARS-CoV-2 (20), while mutations within the ACE2 receptor-binding domain (RBD) of SARS-CoV-2 may reveal an alternative viral-host binding mechanism that can further facilitate viral entry (15). Based on this, SARS-CoV-2 is considered a novel strain that possesses distinct evolutionary paths from SARS-CoV and MERS-CoV and possesses possible lineage similarities to another previously detected bat-derived coronavirus. Differences in genome and proteome profiles highlight their unique immune evasion mechanism and their immunopathology in respect to the host response. In this review, we summarised the immunological features of SARS-CoV, MERS-CoV, and SARS-CoV-2 infection and proposed possible pathogenesis mechanisms by providing supporting evidence based on pre-existing and recent studies.

## SARS: Clinical and Immunological Features

SARS is the first highly pathogenic human coronavirus disease to be identified, and it exhibits a high case fatality rate of 9.6% (21). Based on its short incubation period (mean: 4.6 days) and high transmissibility (Basic reproductive number: 0.95) during the early stages of the epidemic (22) (Table 1), SARS soon triggered a community outbreak, and more than 8,000 cases were reported globally between November 2002 to July 2003. Middle-aged patients ranging from 45 to 55 years were the most prevalent in this pandemic and often presented with typical clinical symptoms of SARS, while lethal pneumonia was more frequently observed among patients over 60 years of age and in immunocompromised individuals. In comparison to MERS patients, SARS patients could develop acute respiratory distress syndrome (ARDS) within the first week of clinical diagnosis and may require a longer time to achieve full recovery; however, disease severity and mortality tended to be milder. According to retrospective studies, advanced age, comorbidities (diabetes, HBV infection), high LDH levels, and high neutrophil and low lymphocyte counts are associated risk factors or indicators for developing severe SARS (Table 1).

Typical SARS infections undergo a clinical course that consists of three phases: the viral replication phase, the immune responding phase, and the terminal phase (3). Each phase is characterised by distinctive immunopathology manifestations and will be introduced in more detail in this review.

### SARS Patients in the Early Phase: Virus Replication

Similar to other viral infections, early SARS infection is characterised by non-specific symptoms such as fever, myalgia (muscle ache), headache, and malaise (severe tiredness). Such symptoms typically resolve themselves within 1 week. Sequential samples of nasopharyngeal aspirates from SARS patients revealed an association between viral load and clinical progression. The viral load surges progressively at early infection and peaks at around day 10, and this peak is delayed compared to those of influenza and RSV infection (3, 23). This peak is followed by a rapid decrease in viral load and by IgG seroconversion, which is an early sign of a shift toward specific immunity. Unexpectedly, the clinical condition of these patients worsens at this time, which is inconsistent with the observations that viral clearance is predominantly occurring. A delayed viral peak can be inferred as delayed or absent effective host anti-viral responses that are necessary for viral clearance. A lack of background immunity in the general population may partially explain the delayed viral clearance that is observed in comparison to that of common respiratory infections. Additionally, the sudden worsening clinical symptoms may indicate other potential invasion strategies that may exist between the host and the virus.

Retrospective studies have revealed an elevation of cytokines and clinical progression following viral load decline, highlighting the underlying pathogenic relationship between immune dysregulation, viral clearance, and disease progression (24). Numerous studies have demonstrated that a hyper-inflammatory response, rather than a viral cytopathic effect, is the primary cause of disease aggravation. Additionally, the rapid elevation in viral loads contributes to the disease pathology to some extent. Early studies observed the presence of viraemia in ~75% of clinically-diagnosed patients at the first week of infection prior to the detection of SARS-specific antibodies (25). Serum viral load has previously been found to be associated with various undesirable events such as oxygen desaturation, diarrhoea, hepatic dysfunction, and death (26), indicating the involvement of high viral loads in organ function deterioration. Interestingly, viral load was also detectable from clinical specimens of various anatomic sites. Among these, stool specimen viral detection was highly correlated with diarrhoea, and viral particles were found to be present in ileum and colonic biopsies under electron microscopy (26). These provide strong evidence supporting the association between high viral loads, viraemia, and pathological effects. It was also speculated that high viral loads orchestrate massive infiltration of pro-inflammatory innate immune cells that could worsen clinical outcomes in patients. Previous studies have also found that SARS patients possessing a high initial viral load were associated with higher eventual mortality (27).

With host entry, the virus initially invades host defences by targeting the ACE2 receptors that are highly expressed on airway epithelial cells. Similar to other coronavirus, SARS-CoV viruses have evolved to encode a vast variety of proteins that could attenuate host anti-viral responses and aid in escape from innate immune responses, and additional strategies such as capping of viral mRNA and replication in double membrane vesicles could aid in escape from host recognition (28, 29). Inhibition of host anti-viral signalling pathways was achieved by regulating immune function-related gene expression by both non-structural and structural viral proteins (30). The targeted pathways include signalling cascades downstream of PRRs, the NF-kB pathway involved in cytokine production, and the JAK-STAT pathway involved in transcription of interferon-stimulated gene (ISGs). Viral proteins can also interfere with normal host cellular functions by modulating the ubiquitination pathway and by degrading host cell mRNA to ensure persistent and productive viral replication (31). Interactions between functional proteins and the host cellular response have been confirmed and extensively discussed in previous reviews (32, 33).

Another important viral entry route involved the infection of haematopoietic cells and PBMC cells, and this has been observed from *in vivo* studies. Infected cells, such as monocyte-derived macrophages and dendritic cells, underwent viral replication in an abortive manner, and this mechanism resulted in the aberrant production of cytokines and chemokines instead of effective viral replication (34). As a result, only incomplete viral RNA possessing a single strand was detected. The cascade activation of chemokines and cytokines could initiate a variety of immune responses and could contribute to the unique immunological profile that is observed in most of these early-infected SARS patients. For example, high levels of CXCL-10/IP-10, CCL-2/MCP-1, CXCL-5/RANTES, and CCL-3/MIP-1α enhance the recruitment of dendritic cells and macrophages to the site of infection (35, 36). CXCL-10/IP-10 and CCL-2/MCP-1 could suppress haematopoietic progenitor cells proliferation, ultimately leading to lymphopenia (37). The pro-inflammatory cytokines IL-8, TNF-α, and IL-6 induce migration and recruitment of neutrophils, pro-apoptotic T-cells, and epithelial cells (38, 39). Unexpectedly, most SARS patients experience a robust upregulation of type-I interferon during the early phase of infection that occurs concurrently with an upregulation in the expression of IFN-stimulated chemokines (CXCL-10 and CCL-2). However, such immune responses were soon restored to a neutral level and were regulated in non-severe and recovered patients (40).

A downregulation of cytokines was also observed in these infected cells, and a reduction in IL-12 expression was observed *in vivo* in dendritic cells to further suppress the conversion of the Th1 cell phenotype, which is an essential type of cell-mediated immune response that is involved in viral clearance. Atypical up-regulation of cytokines and chemokines due to abortive infection of dendritic cells can imbalance the induction of T-helper cell subsets (35) by affecting the migrating and antigen-presenting function of dendritic cells (DC) to ultimately skew T-cell activation.

### SARS Patients in Crisis Phase: Cytokine Storm

Most patients proceed to the second phase after 7–10 days of the asymptomatic period. A sudden recurrence of fever accompanied with respiratory symptoms is often the relapse manifestation. Signs of clinical progression include diffuse ground-glass opacity found on chest CT scans and the development of acute respiratory distress syndrome (ARDS) that presents as worsening dyspnoea and a dramatic decline in arterial blood oxygen saturation. Approximately 20% of patients enter the terminal phase where they develop critical conditions such as multiple organ dysfunction syndrome (MODS), severe lymphopenia, and nosocomial sepsis (3, 41).

This is typically the phase in which major pathological incidents have taken place. The magnitude and variety of the host immune response that is activated correlates with disease severity and outcome. Initially, host immune defence mechanisms are comprehensively upregulated to a functional state to achieve effective viral clearance. However, failure in maintaining immune homeostasis against the multifaceted viral invasion and evasion strategies could lead to dysregulated immune responses that result in massive pathophysiological consequences, ultimately leading to disease deterioration. For patients experiencing mild infection, successive host immune responses were induced in respect to the severity of infection, and these patients were capable of regulating or shifting to adaptive immunity when confronted with extensive viral invasion. In addition to the comprehensive anti-viral strategies elicited, these patients soon achieved clinical recovery without experiencing disease aggravation. Immunopathology impacts involve atypical manifestations of immune cell responses that could weaken viral clearance efficiency and could augment pathological effects. Either dysregulation in the production of cytokines/chemokines or imbalances between innate and adaptive immunity could increase the risk of disease progression in hosts.

A study of cytokine profiles in a severe SARS group revealed a substantial elevation of chemokines (MCP-1, MIP-1α, IP-10, IL-8) and pro-inflammatory factors (IL-1, IL-6, IL-12, TGF-β, and INF-α) (34), while a less intense activation of innate immune-related cytokines was observed in recovered patients (Table 2). Additionally, Th2-related cytokines (IL-4, IL-5, IL-10) were significantly increased in deceased SARS patients (44), and such imbalances in Th1/Th2 cytokines were also typically observed in influenza-infected elderly patients who represent a high-risk patient group with increased case fatality rates (55). This raises the possibility of the contribution of age to disease outcome. In contrast, a marked elevation of Th-1 related cytokines, interferons, and other cytokines (IL-2, IL-12, IFN-γ, TNF-α) was found in mild patients. This Th-1 cell phenotype is pivotal in mediating virus-specific adaptive immunity and, together with sufficient anti-viral interferons, it promotes the phagocytic activity of macrophages and stimulates the proliferation and activation of cytotoxic T lymphocytes (CTLs) to allow for effective and specific viral clearance (42). It is important to note that the study of cytokine production in clinical patients requires dynamic monitoring and a large study population. The observed results from a series of studies may possess large variations that can be attributed to study design (56) (Table 2). This is due to the observation that the detection of cytokine levels largely depends on the phase of clinical course, disease severity, types of specimens collected, types of cytokines assessed, the detection method used, and previous medications. It is difficult to provide an absolute conclusion about the cytokine profiles of SARS patients. However, studies examining cytokines provide clues to the type of immune response or pathophysiological events involved in this disease and could provide a clearer picture of SARS pathogenesis.

**Table 2:** Cytokines and chemokines changes in SARS, MERS, and COVID-19 infection during different phases of disease course.

**Types of viral infection and study**	**Study method, subjects and size**	**Elevation of chemokines**	**Elevation of inflammatory cytokines**	**Down expression of cytokines or fail to observe changes**	**Implication for cytokines profile observed**	**References**
**1. Early phase or acute phase**						
SARS (*in vivo*)	20 non-severe SARS patients had their serum cytokines consecutively measured for 19 days	MCP-1, IL-8, IP-10	IL-6, IL-1β, IL-12, IFN- γ	TNF-α, IL-10, IL-2, IL-4	Th-1 related cytokines were significantly increased and induced a hyperinnate inflammatory response. IP-10 was chemoattractant of monocytes, T cells, and NK cells, responsible for inflammatory cell infiltration	(42)
SARS (*in vivo*)	88 hospitalised SARS patients had their serum cytokines dynamically measured in the first 20 days of infection	MCP-1, MIG, IL-8, IP-10	IL-6, IFN- γ, IL-18, TGF- β	TNF-α, IL-10, IL-2, IL-4, IL-13	IFN-γ-related inflammatory cytokines were already elevated at early infection	(43)
SARS (*in vivo*)	Serum obtained from 98 acute SARS patients within 2 days of hospital admission	IL-8	IFN-γ, IL-6, IL-10, IL-12	/	Cytokines were mainly produced by monocytes and NK cells	(44)
SARS (*in vitro*)	Human mononuclear cells isolated and cultured to induce dendritic cells, later infected with SARS-CoV and cytokines were quantified by real time RT-PCR at 3 and 9 h after infection	MIP-1α,RANTES, IP-10, MCP-1	TNF-α, IL-6	IFN-α, IFN-β, IFN- γ, IL-12	Moderate upregulation of cytokines (TNF-α, IL-6) and significant upregulation of chemokines was observed, which might be responsible for migration of inflammatory cells and facilitate viral spread. While low expression of anti-viral cytokines (interferons) might involve mechanisms of immune evasion	(35)
MERS (*in vivo*)	Serum from 7 mild MERS patients were obtained within 2 days of hospital admission and was compared with healthy controls	/	IFN-α2, IFN-γ, TNF-α, IL-15, IL-17, IL-10	IL-2, IL-4, IL-5, IL-13, TGF-α	A prominent pro-inflammatory Th1 and Th17 response was observed. IL-17 could recruit monocytes and neutrophils to sites of infection and enhance production of Th17-related cytokines. Induction of IFN-γ and IFN-α2 could promote antigen presenting and antiviral Th1 response. Elevated IL-10 might play a role in host immune regulation. No elevation in IL-12 and Th-2 cytokines was observed	(45)
MERS (*in vitro*)	Polarised airway epithelial Calu-3 cells were infected with MERS-CoV and SARS-CoV and cytokines were quantified within 30 h of infection	IL-8	IL-1β, IL-6	TNF-α, IFN-β, IP-10	In comparison to SARS, pro-inflammatory cytokines were markedly elevated in a delayed manner, while no significant induction of anti-viral cytokines were observed. This suggests a delayed pro-inflammatory and attenuated anti-viral response in MERS infection	(46)
MERS (*in vitro*)	Monocyte-derived macrophages were inoculated with MERS-CoV, supernatants and cell lysates were harvested at several time points post-infection for cytokine measurement	IP-10, MCP-1, MIP-1α, IL-8, RANTES	TNF-α, IL-6, IL-12, IFN- γ	/	Chemokines and cytokines were induced in a delayed manner, however, presented at a higher magnitude (IL-12, IFN- γ and chemokines) and prolonged intervals than SARS	(47)
COVID-19 (*in vivo*)	40 patients (13 severe and 27 mild) had their serum cytokines and lymphocytes subsets dynamically measured in the first 16 days of infection	/	IL-4, IL-10, IL-2, IFN-γ, TNF-α	/	T cells are essential in attenuating overactive innate immune responses. Kinetic changes of T cell counts are negatively correlated to that of cytokines. This significant decrease in T cells might result in aggravated inflammatory response in COVID-19	(48)
COVID-19 (*in vivo*)	41 patients (13 ICU and 28 non-ICU)	IP-10, MCP-1, MIP-1α, GSCF	IL-2, IL-7, IL-10, TNF-a, IFN-γ, IL-1β	/	ICU patients had higher levels of cytokines. IL-1β, IFN- γ, MCP-1, and IP-10 could lead to activated Th1 responses. Both Th1 and Th2 cytokines were observable in COVID-19 patients	(49)
**2. Crisis phase or terminal phase**						
SARS (*in vivo*)	Serum obtained from 98 acute SARS patients within 2 days of hospital admission, in which 11 patients died	/	IL-4, IL-5, IL-10	/	Significant increase of Th-2 cytokines was observed in fatal cases. Imbalance of Th1/Th2 cytokines was also observed from elderly patients with influenza infection, suggesting this might be a key influence in the outcome of the elderly	(44)
SARS (*in vivo*)	Serum were obtained from 3 groups of SARS patients graded as mild (*n* = 30), severe (*n* = 30), and convalescent (*n* = 30) SARS. Serum cytokines were measured at single time point	/	IL-6	TGF- β, IL-8, TNF- α, IL-1 α	Decrease in IL-8 and TGF- β may be consistent with T lymphocytes depletion in severe patients. While decrease of T lymphocyte is associated with severity of SARS. Levels of TNF- α and IL-1 did not differ between SARS and control, this was inconsistent with results in influenza infection and suggested the need for cytokine detection in bronchoalveolar lavage fluid	(50)
MERS (*in vivo*)	Serum obtained from 2 distinct outcome MERS patients dynamically	IP-10	IL-10, IL-17	IL-12, IFN-γ	High levels of IP-10 were associated with persistent viral replication. Lack of IFN- γ and IL-12 lead to ineffective in developing Th-1 response. Elevation of IL-10 further suppress IFN- γ production and was associated with poor outcome. High levels of IL-17 were also observed in fatal patient	(51)
COVID-19 (*in vivo*)	53 patients (34 severe and 19 mild) had their blood plasma collected at the earliest time-point after hospitalisation and serum cytokines were measured	IP-10, MCP-3	IL-1ra	/	IP-10, MCP-3, and IL-1ra were independent predictors for COVID-19 progression. Combination of the 3 cytokines showed biggest AUC of the ROC calculation, associated with disease deterioration and fatal outcome	(52)
COVID-19 (*in vivo*)	548 patients (269 severe and 279 non-severe) had their serum cytokines measured at admission	/	IL-2R, IL-6, IL-10, TNF- α	/	Th-1 cytokines (IL-6, TNF- α) were significantly elevated in severe cases, similar to results observed in SARS infection	(53)
**3. Convalescent phase or recovery**						
SARS (*in vivo*)	Serum obtained from 88 SARS patients during convalescent phase (30 days or later post disease onset) was compared with serum from their acute phase	/	/	IFN- γ, IL-18, TGF- β, IL-6, IP-10, MCP-1, MIG, IL-8	All of the elevated cytokines in the acute phase were normalised returned to basal level, in which statically significant decrease of IFN- γ and IL-6 were observed	(43)
SARS (*in vivo*)	Serum were obtained from 3 groups of SARS patients graded as mild (*n* = 30), severe (*n* = 30), and convalescent (*n* = 30) SARS. Serum cytokines were measured at single time point	/	IL-10	IL-4, INF-γ	Levels of Th2 cytokines were altered compared to normal controls. IL-10 was known to inhibit TNF- α production and neutrophil activation. Thus, increased IL-10 may reflect some protective mechanisms	(50)
MERS (*in vivo*)	Serum obtained from 27 MERS patients during convalescent phase (period immediately after the negative conversion of real-time RT-PCR) was compared with serum from their acute phase	/	RANTES	IL-6, IL-1RA, IP-10, MCP-1	Levels of cytokines was proportionally related to disease severity. Elevated cytokines (IL-6, IL-1RA, IP-10, MCP-1) observed in acute phase declined to basal level at convalescent phase. Elevation of RANTES in convalescent phase might be associated with activated virus-reactive T lymphocytes	(45)
COVID-19 (*in vivo*)	Dynamics of peripheral immune cells, cytokines, and HLA-G and its receptor expression in a COVID-19 patient at convalescent stage	/	IL-4, TNF-α	IL-6, IL-10, IFN-γ	Dynamic HLA expression and cytokine expression from SARS-CoV-2-positive to SARS-CoV-2-negative status indicated that regulation of HLA-G expression is involved in SARS-CoV-2 infection, which might impair CD8+ CTL mediated recognition and support immune evasion	(54)

For all cytokines, the activation of upstream inflammatory signals, such as IL-17 and IFN-γ, exerts a significant effect in regard to orchestrating cytokine production and activating multiple immune cells (43, 57), and this is associated with disease severity. IL-17 is a T helper cell-derived cytokine involved in autoimmune disease and viral infection (58), and this cytokine is capable of orchestrating broad pro-inflammatory responses. Elevation of IL-17-related cytokines has been observed in human coronavirus infection both *in vivo* and *in vitro* (45, 59). In post-SARS patients, IFN-γ-related cytokines (IL-6, IL-8, IL-18, TGF- β, MCP-1, MIG, and IP-10) were significantly elevated in the fatal group compared to levels in the survival group. Such elevation was not observed in the mild phases and was demonstrated to be independently correlated to levels of IFN-γ. Chemokines such as IP-10 are potent chemoattractants of activated cytotoxic T lymphocytes (CTL), natural killer cells, and monocytes. IP-10 can facilitate the infiltration of inflammatory monocyte-macrophages (IMM) into the lung interstitium and the alveolar space (56), and this chemokine appears to be a critical factor causing the exacerbation of ARDS (60). MCP-1 and IL-8 were also demonstrated to be robust inducers of monocytes. IL-18, IL-6, and TGF-β possess pro-inflammatory effects and are responsible for lung tissue injuries and necrosis. Additionally, an IFN-γ-mediated cytokine storm is associated with disease aggravation and poor clinical outcome (43).

### SARS Patients in Crisis Phase: Dysregulated Immune Response

The stimulation of B cell immunity and the secretion of neutralising antibodies (nAb) is associated with a rapid decline in serum viral load; however, these processes are not related to disease resolution (3). Instead, antibody-dependent cell-mediated cytotoxicity (ADCC) and abortive infection may be the underlying mechanisms of viral depletion; however, both of these processes could further enhance the magnitude of cytokine storms, thus acting as a double-edged sword with disease outcome. In severe patients, deviations in ISG and immunoglobulin gene expression, sustained release of cytokines, and impaired production of antibodies were observed (40), suggesting the presence of a dysregulated IFN-mediated innate immune response and a suppressed level of cellular immune response. Interferons orchestrate both innate and adaptive immunity via interacting with the corresponding signalling pathways through their functional interferons receptors (IFNR) (61). The inability to attenuate innate immune responses and the development of early effective adaptive immunity to achieve viral clearance would lead to augmented pathological effects. Additionally, the ability of the virus to evade host immune surveillance and to interact with molecules of the JAK-STAT signalling pathway could result in diminished levels of interferons, ultimately reducing the anti-viral responses required to contend with the exaggerated inflammatory responses (62). Delayed IFN signalling in combination with the pro-inflammatory and chemotaxis effects from surging viral titres could lead to excess accumulation of inflammatory monocyte-macrophages (IMM) at infected sites, where these cells could again produce excessive inflammatory cytokines and oxidative stress-related molecules to induce a cycle of IMM infiltration and extensive lung injuries (36, 63). Delayed IFN-induced cytokines could also alter T cell activation. In severe patients, the activation of CD8+ T cell responses was significantly stronger and more frequently observed. The degree of CD4+ T cell responses primarily depends upon the number of epitopes recognised (primarily found in spike proteins) and correlates with disease severity. Further investigation revealed the presence of a poly-functional memory CD4 + T cell phenotype (producing IFN- γ, IL-2, and TNF- α) in the severe group that may be involved in the pathogenesis of this disease. In recovered patients, neutralising antibodies and T cell responses specific to spike proteins (primarily CD4+ T cells) were most commonly found (44).

### SARS Patients in Crisis Phase: Antibody Response

Regarding the observed dynamic changes of antibody titres in SARS-recovered patients, most patients experience seroconversion by day 16 of infection, while virus-specific IgG and nAbs peak at 4 months post-infection and decrease markedly at 16 months (64). A long-term follow-up study revealed that specific Abs gradually decline, and the presence of specific IgG was only found in sporadic cases (two out of 23 recovered patients) at 6 years post-infection. These recovered patients that were unable to elicit peripheral memory B cell responses did possess active memory T cell responses that were extensively detected (14 out of 23 recovered patients). This suggested a predominating role of memory T cell in providing long-term memory in recovered patients and raised concerns regarding the protective function of antibodies in SARS infection (65).

Unexpectedly, the presence of IgG could be detected soon after the onset of symptoms (< day 16) in severe SARS patients. Higher titres of IgG in the acute phase were more frequently observed in patients that required supplemental oxygen and ICU admission. These findings again raise questions regarding the possible role of antibodies and their relation to disease severity during SARS infection (66). However, unlike the sustained levels of nAb activities that could last for as long as 300 days in recovered patients, deceased patients exhibit a rapidly diminished antibody response soon after initial activation. The excessive over-reactive immune response observed in deceased patients may contribute to the systematic breakdown of the host immune system, ultimately failing to provide long-term protective memory (67).

One study verified the detrimental role of SARS antibodies on deviations in macrophage function. Anti-spike protein IgG induced a lower wound-healing effect on macrophages, and this was potentially modified through interaction with FcγRs. Elevations in IL-8 and MCP-1 could further trigger the recruitment of monocytes/macrophages and lead to intense inflammatory pathological changes (68). Another *in vitro* study proposed the existence of a possible signalling pathway involved in the polarisation of macrophages. Upregulation of STAT-1 is necessary for interferon signalling pathway function, while a STAT-1-related pathway is also independently involved in the alteration of macrophage phenotypes. A previous study demonstrated that inhibition of the STAT-1 pathway not only leads to delayed production of IFN (69), but is also related to shifting of the macrophage function. A reduction in STAT-1 could also promote the alternative activation of macrophages and result in lung injuries (70). This immunological feature is also characteristic of elderly patients and may contribute to the pulmonary fibrosis commonly found in elderly SARS patients (71). Additionally, although antibody-mediated cytotoxicity may promote viral clearance, the accompanying augmented pro-inflammatory effects in terms of continuous IMM infiltration result in greater detrimental effects to the host (68).

The role of antibodies in maintaining adequate long-term protection has long been questioned due to distinct performances observed in mild/severe recovered patients and inconsistent results related to vaccine development (72). Antibody-dependent enhancement (ADE) is another pathological mechanism that has been found in flavivirus and other coronaviruses, where antibodies could enhance viral entry via interaction between viral protein, IgG Fc segment, and host immune cell receptors (73). Prior immune response to CoV and pre-existing sera antibodies targeting a distinct serotype could mediate such viral-cell entry effects rather than causing neutralisation of the virus, and this could further enhance viral shedding and raises concerns in regard to vaccine design (74). Investigations examining vaccine efficacy have revealed the potential of immune serum to triggering an ADE effect, and this emphasises its dependency on the type of immunisation strategy utilised (75). Additionally, a recent study examining MERS-CoV focused on the molecular structure of monoclonal antibodies and proposed a novel ADE mechanism (76).

### Other Immunological Manifestations in SARS Patients

Early induction of T cell-mediated responses (especially CD8+ T cell) is essential for host survival from lethal infection (77). However, innate immune components that are essential for T cell response were not found to be activated in SARS infected cells, suggesting a failure in mounting the protective T cell response. Later, researchers were able to elicit potent T cell responses *in vitro* by promoting enhanced dendritic cell migration and activation (78). Even in the absence of an innate immune response, the protective effect of virus-specific T cell responses resulted in host survival and clinical improvement (79). The fast response and specific targeting nature of T cells could facilitate effective early viral clearance. Consistent with the results from recovered patients, a persistent memory T cell response was more prevalent than the memory B cell response, suggesting a possible long-term protective role (65). Additionally, the induction of early protective cytotoxic T lymphocytes (CTL) or airway memory CD4+ T cells could be achieved in animal experiments via immunisation strategies and was demonstrated to provide host protection (79, 80). These findings demonstrated the protective role of T cell immunity and provided implications for T cell-based vaccine development (81).

During SARS infection, the expression of lymphocytes is characterised by a significant reduction in CD4+ and CD8+ T cells subsets, which is commonly observed as lymphopenia (82). The activation and differentiation of naïve T cells largely depends on costimulatory signals, and any of these extrinsic defects would contribute to T cell inactivation. In addition to the unfavourable impacts caused by aberrant activated cytokines during the innate immune response, age-related defects in respiratory dendritic cells (rDCs) is another hypothetical mechanism that could result in impaired antigen processing and migration properties. The expression of CCR-7, a chemokine binding receptor involved in DC migration, was down-regulated during SARS infection, and this down-regulation could affect the drainage of rDCs to lymph nodes. Further investigation revealed that elevated expression of prostaglandin D2 (PGD2) was exclusively found in the aged SARS group and could further suppress CCR7 expression to possibly indirectly lead to the impairment of rDCs. In SARS settings, PGD2 was significantly synthesised and accumulated, and based on this, such defects would result in delayed T cell priming and differentiation of effective CD8+T cell responses (83).

Other potential mechanisms of lymphopenia have been widely reviewed. A lack of costimulatory signals from antigen presenting cells was previously suggested. An imbalanced Th1/Th2 cell response may also mediate an unusual cytokine profile leading to T cell inactivation, and initiation of excessive adaptive immune responses could lead to early T cell exhaustion and a breakdown of the immune system. Pro-apoptotic effects elicited from viral structural proteins, altered IFN-I levels, and glucocorticoid therapy are other possible contributors to T cell apoptosis and lymphopenia (44, 84).

## MERS: Clinical and Immunological Features

MERS (Middle East respiratory syndrome) is the most lethal human coronavirus infection to be identified so far. Although is exhibits a lower transmissibility rate among humans, nearly all MERS-CoV infections can result in severe symptoms, and these infections are challenging in regard to clinical management. MERS-CoV was later found to be primarily circulated only among camel populations, and thus, community outbreaks tended to be in small clusters between families or friends who were in close contact with primary infected individual (4). Similar to SARS patients, MERS patients initially present with mild symptoms followed by rapid development of dyspnoea and respiratory failure in post stages. In contrast, more than half (63.4%) of MERS patients ultimately develop lethal pneumonia. Organ function later deteriorates in a more rapid manner and can lead to fatality as early as 2 weeks after infection (Table 1). Comorbidities such as renal failure and diabetes were more prevalently found in MERS patients and are known as risk factors for poor outcome (Table 1) (85). In this section, we focus on comparing the pathogenesis and host-viral effects between SARS-CoV and MERS-CoV infections, and we emphasise the distinct immune features observed in MERS-CoV infection.

### Different Immunopathological Effects

Unlike the abortive infection mechanism found in SARS-CoV, MERS-CoV could replicate effectively in macrophages, dendritic cells, and lymphocytes (47, 86, 87). This was confirmed by detection of nucleoprotein expression, viral genomes, and viral particles in those infected cells. Productive viral replication in dendritic cells and macrophages indicated that host cells could serve as viral reservoirs and shield the virus from host immunorecognition (87). Additionally, these infected cells could enhance systemic dissemination and facilitate the spread of the virus to lymph nodes, thus allowing the virus come into contact with naïve T cells and initiate robust adaptive immune responses that lead to the production of extensive levels of cytokines and chemokines. This diverse activation avenue in triggering cytokine production during MERS infection clearly results in a unique cytokine profile that is different from that observed in response to SARS infection. Productive replication may be due to the high levels of DDP4 receptors expressed in monocytes and dendritic cells compared to the low levels of ACE2 receptors targets during SARS infection (88), ultimately leading to differential infection outcomes. Additionally, MERS-CoV was capable of infecting cells derived from various human cell lines *ex vivo* (89). DDP4 receptors were also abundantly found in epithelial and endothelial cells of the liver, kidney, intestines, and prostate (17, 90, 91). These findings support the clinical evidence that viral dissemination was more commonly observed in MERS infection and explain the high incidence of systemic events, such as septic shock and multi organ failure (4).

Antibody dependent enhancement (ADE) is another immunopathological feature that has been confirmed in MERS. Recently, an enhanced membrane-fusion process was identified as the underlying mechanism. It is suspected that interactions between antibodies and the RBD of the S protein could increase the proteolytic susceptibility and lead to conformational changes in target cells. This occurs after binding of nAb and immediately guides the entry of the virus through the canonical receptor-dependent pathway (76).

### Different IFN-I Effects

SARS-CoV possesses an established comprehensive IFN antagonism, while MERS-CoV may employ less effective antagonist activity that may result in increased sensitivity to IFN-I anti-viral responses (92). As it is known to share similar viral interferon antagonism and evasion strategies with SARS-CoV (33), MERS-CoV could suppress the upregulation of antiviral ISG responses via a novel approach that is independent of interferon signals and instead directly induces repressive histone modifications in host cells (93). This altered histone modification was also found in response to H5N1 infection and could mediate a variety of biological events, such as gene regulation. By modifying the basal state of host chromatin, genes are packed in a close conformation that would inhibit the binding of transcription factors (94). This mechanism could explain the low expression of ISGs that was observed in interferon-administrated MERS infected mice (95).

Delayed elevation of endogenous IFN-I is known to be a direct consequence of viral evasion and interferon antagonism, and it often leads to detrimental events (61). Similar to SARS-CoV infection, delayed and attenuated levels of IFN-I were also observed in response to MERS-CoV infection (46). As previously observed in a SARS experiment, the absence of IFN-I caused no significant lung immunopathology and instead improved clinical outcome when compared to that of the delayed IFN-I group, suggesting an atypical IFN-I effect in the context of SARS infection (63). Surprisingly, adverse results were observed in MERS infection scenarios. Early administration of IFN-I could protect mice from lethal infection despite the down-regulation of ISG and cytokine-related genes. Delayed or absent IFN-I responses resulted in no improvement in clinical outcome. It was suggested that recruitment of activated IMM, a dysregulated pro-inflammatory cytokine response, and the inhibition of optimal virus-specific T responses were the underlying mechanisms. In addition to the anti-inflammatory immune-modulatory role during the acute phase, IFN-I signalling may provide benefits by mediating an earlier adaptive immune response during acute MERS infection (95). When determining the role of IFN-I in the context of MERS, it is necessary to consider the relationship between viral replication kinetics (maximum replication) and relative IFN-I response timing.

### Different Adaptive Immune Responses

In critically ill MERS patients, a failure in the activation of Th1 cells often leads to reduced IFN-γ production, and this could affect the activation of CD8 + T cells and NK cells and result in attenuated viral clearance and uncontrolled immune response. While persistent secretion of IL-10 could attenuate the Th1 cell response in SARS patients, high levels of IL-10 in relation to the Th2 cell immune response were also associated with poor outcomes (44, 51). These observations further emphasised the importance of inducing the Th1 cell immune response during the early phase in MERS patients. Another study analysed and detected levels of MERS-CoV-specific CD4+ and CD8+ T cell responses and cytokine expression in MERS patients who had recovered. Neutralising antibody (nAb) titres were found to be strongly correlated with levels of CD4 + but not CD8+ T cell responses in critically ill patients, and this was possibly due to the persistent viral shedding and prolonged interaction for antibody production (96). In contrast, survivors that have experienced mild infections develop prominent levels of virus-specific CD8 + T cell responses without detectable antibodies, indicating that efficient and rapid virus clearance may have occurred prior to CD4+ T cell responses and antibody-production. Although the induction of CD8+ T cell responses could facilitate viral clearance at the acute phase, robust inflammatory and cytotoxic T lymphocyte (CTL) responses could potentiate lung pathology and exhaust host immunity. In another study, extraordinary robust CD8+ T cell responses were observed in severe patients at the acute phase, suggesting that hyper-activated T cell responses were not beneficial (97). Despite this, the importance of the acute phase T cell response is an agreement with results that were previously observed in SARS patients. Activation of the T cell response in the absence of the innate response was sufficient to enhance host survival and resulted in disease improvement (77, 79). This further suggested that the advantages of an early initiation of disease-proportional adaptive immunity might outweigh any disadvantages in the context of MERS infection.

Another study proposed an evolved mechanism found in MERS that involved the down-regulation of antigen presenting pathway-related genes, including type I and II major histocompatibility complex (MHC) pathway genes and components of the immunoproteasome. Reduced expression of MHC molecules attenuated CD8+ T cell-mediated recognition of infected cells and allowed for viral evasion. Diminished presentation of antigens further led to imbalanced Th1/Th2 cell activation that could alter the ability to mount an effective adaptive immune response (59). In contrast, earlier upregulation of MHC-related genes was observed in another study (47). These diverse results indicate that adaptive immunity during MERS infection does not always elicit a functional effect. It is essential to consider the activated components of adaptive immunity. In conclusion, the adaptive immune response observed in the acute phase of MERS may contribute to a more positive outcome than that observed in SARS (96).

### Different Cytokine Storms

Among all cytokines, the expression of IL-17 was more significantly upregulated compared to that in SARS infection (45). IL-17 is secreted by CD4 + T cells and can orchestrate robust and deleterious pro-inflammatory effects on host cell. The expression of IL-17 has been confirmed to exacerbate respiratory syncytial virus (RSV) and seasonal influenza infection. In the context of MERS, IL-17 is believed to induce immune-mediated pathology and to contribute to elevated mortality rates (59). In comparison to cytokine profiles in SARS infection, similar types of cytokines were produced in a delayed manner. MERS infection is characterised by a relatively higher and prolonged secretion of these cytokines (Table 2) (47). In acute phase patients, higher levels of pro-inflammatory cytokines (IL-6, IL-8, and IL-1β) and attenuated antiviral cytokines (TNF-α, IFN-β, and IP-10) were observed when compared to those in SARS infection (46). However, in some rare conditions, the extensive induction of innate pro-inflammatory cytokines in the absence of a T cell response during the acute phase may orchestrate a cytokine storm and result in fatality (97).

## COVID-19: Clinical and Immunological Features

The recently discovered human coronavirus that emerged in late 2019, SARS-CoV-2 (2019-nCoV), has resulted in the most devastating pandemic of the twenty first century, and this virus has infected more than 10 million patients globally due to its high transmissibility and viral shedding properties (Table 1) (2). COVID-19 patients often present with varied flu-like onset symptoms that can include fever, myalgia, and dry cough. Mild manifestations and asymptomatic infections are common among immunocompetent individuals and children, and these individuals represent the main infected source during this pandemic outbreak. In contrast, the elderly and immunocompromised individuals often develop lethal symptoms. These distinct differences suggest that different pathogenesis may exist between age groups.

Dynamic changes in serum leukocytes were detected in all COVID-19 patients and persist throughout the course of the disease. A slight decrease in total leukocyte counts in mild patients may result from transient lymphocyte exhaustion (48). In critically ill patients, an early elevation in leukocytes and neutrophils in response to pro-inflammatory cytokines was observed (9). In addition to inducing innate immune responses and activating cascades of cytokine signalling pathways (IL-6, IL-1β), these pro-inflammatory cytokines function in conjunction with virus-damaged endothelial cells to mediate neutrophils to perform viral clearance via distinct approaches. NETosis is an oxidative pathogenic mechanism that involves the tremendous release of oxidant enzymes such as MPO, NADPH oxidase, and nitric oxide synthase at the extracellular space. This mechanism is primarily utilised by neutrophils to contain and eradicate pathogens that would later propagate extensive inflammation and form microvascular thrombosis if not properly activated, ultimately leading to diffuse lung tissue injuries (98). Peripheral lymphocytes exhibited significant depletion and an alteration in subsets (99), and this often resulted from dysregulated innate immune responses or from functional exhaustion induced by the expression of the T cell inhibitor “checkpoint” receptor (100). These processes are all important in the context of viral clearance, immune dysfunction, and disease progression. A retrospective study revealed that 83.2% of patients had at least once manifested lymphopenia during the acute phase of infection (10). Lymphopenia is clearly one of the more prominent features found in COVID-19 infection and may represent an associated factor to disease severity.

### COVID-19 and Innate Immunity

**Figure 1:**
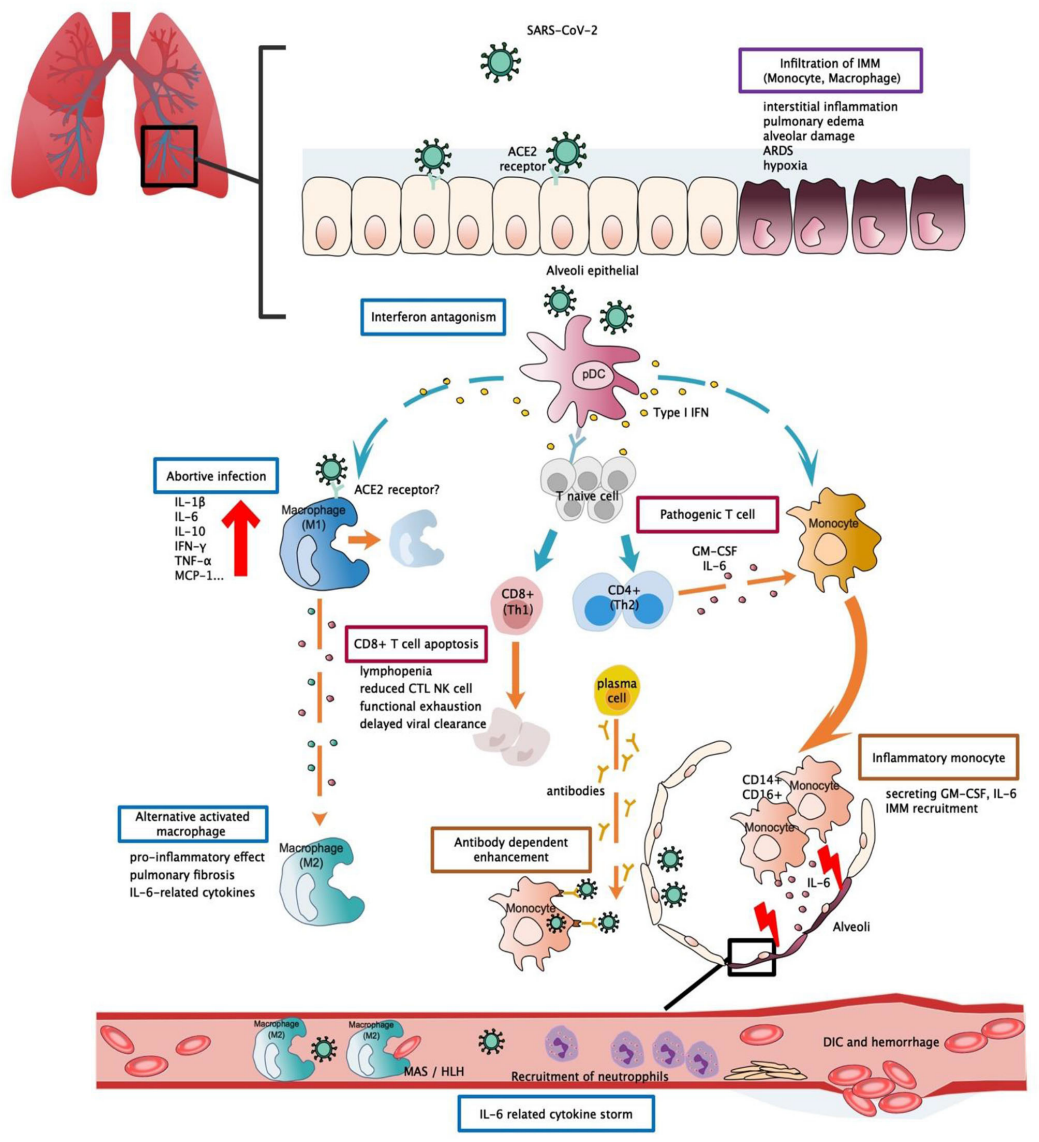
Potential immunopathogenesis in SARS-CoV-2 infection. This figure shows the potential immunopathogenesis during SARS-CoV-2 infection, inferred from previous SARS-CoV and MERS-CoV studies. Coloured boxes labelled the potential strategies or deleterious events involved in SARS-CoV-2 pathogenesis. Words below each box indicate the pathological consequences. Dashed arrows indicate causal relations between target cell and cell mediators. (A) Initially host-viral entry was found at alveoli epithelial. The virus invades host defences via binding with ACE2 by S-protein RBD. Abortive infection was observed in PBMC and haematopoietic cells—a process that induces expression of pro-inflammatory mediators rather than effective viral production. Another potential viral entry strategy relies on the presence of specific antibodies that form bridges between viral-host and facilitate viral entry rather than expressing ADCC effect. SARS-CoV-2 might have evolved to encode specific proteins to counteract the host anti-viral response and optimise viral entry. Strategies such as interferon antagonism (not shown on the figure) allow viral evasion and prolonged viral shedding. (B) Regarding the host immune response, increased viral loads, and chemokines from abortive infection further enhance infiltration of IMM, an intense release of inflammatory cytokines that results in lung tissue injuries. Delayed viral clearance, aberrant cytokine production, and altered interferon levels hinder the proper functioning of the immune system, such as shifting of functional phenotype in macrophages and lymphocytes which would result in the impaired wound-healing function T cell apoptosis, pathogenic T cell response, functional exhaustion, dysregulated cytokine storm (i.e., MAS/HLH) and impaired viral clearance. Cascades activation of cytokine and chemokine ultimately led to systemic cytokine storm, manifested as sepsis, DIC, haemorrhage, and shock. RBD, receptor binding-domain; ADCC, antibody-dependent cell-mediated cytotoxicity; ACE2, Angiotensin-converting enzyme 2; pDC, Plasmacytoid dendritic cell; IMM, Inflammatory monocyte/macrophage; MAS, macrophage activation syndrome; HLH, Hemophagocytic lymphohistiocytosis; DIC, Disseminated intravascular coagulation.

One of the pathological findings from COVID-19 fatal cases is the presence of an increased infiltration of inflammatory cells within lung tissue (101). This was also confirmed by analysis of the bronchoalveolar fluid (BALF) of COVID-19 patients during infection. An increased proportion of monocyte-derived macrophages were found in BALF, and this proportion was as high as 80% of all infiltrated cells when observed in severe patients. Monocyte-derived macrophages were observed to exist in diverse activated forms. Within the BALF, highly inflammatory macrophages with potent chemokine producing effects were observed (102). Expression of surface ACE2 receptors, the established entry binding receptors of SARS-CoV-2, was detectable in alveolar macrophages, suggesting a possible entry avenue for the virus. These findings provide sufficient evidence for the central role of monocytes in cytokine storm and lung pathology (103) (Figure 1). It should be noted that a failure to shift macrophages from a pro-inflammatory classically activated phenotype (M1) to a wound-healing alternatively activated phenotype (M2) could contribute to the excessive inflammatory injuries and fibrosis lesions commonly found in ARDS patients. This phenomenon has been evaluated in detail in the context of SARS research and is in agreement with the results observed from SARS-CoV-2 patients (68).

Monocytes are capable of differentiating into macrophages and dendritic cells when activated by the innate immune response and are abundantly found in the serum and throughout the circulation. Although no significant change in serum monocyte levels was observed in COVID-19 patients (48), morphological changes and the expression of inflammatory-related phenotypes in monocytes may be involved in disease aggravation. Flow cytometric analysis detected higher levels of IL-6, IL-10, and TNF-α from morphologically different monocytes collected from PCMB of severe patients, thus supporting the presence of an inflammatory monocyte phenotype and a participating role in cytokine production (104). In another study, it was hypothesised that hyper-activation of pathogenic Th-1 cells may generate extensive IFN-γ and granulocyte-macrophage colony-stimulating factor (GM-CSF) signals. Monocytes, which function as the responsive cells of pathogenic GM-CSF, are activated and converted into high levels of CD14 + CD16 + inflammatory monocyte subsets in infected patients. These atypical monocytes can enter the pulmonary circulation and are capable of secreting high levels of GM-CSF+ and IL-6 to further induce monocyte migration and mediate the infiltration of inflammatory macrophages and dendritic cells, ultimately leading to aggravating lung injuries (105) (Figure 1). Moreover, a recent paper reported that high levels of ACE2 entry receptors were expressed on CD68+ and CD169+ macrophages that were found in the spleens and lymph nodes of COVID-19 patients (106), and this detection occurred concurrently with the detection of viral nucleoproteins. Anti-viral signalling molecules, such as IFN-α and IFN-γ, may result in increased expression of ACE2 receptors (107). However, the precise role of such an interferon response remains unclear, as IFN-I was observed to possess diverse functions during SARS and MERS infections (63, 95) that largely depend on the relative timing of viral replication and the virulence of the virus. These findings provide a possible role for the activated T cell response about macrophage activation during acute infection.

The induction of monocyte subsets with altered signatures has been demonstrated to play a critical role in contributing to the occurrence of cytokine storm at the acute phase, as this induction leads to atypical functioning of macrophages and other immune cells. Some of these proposed pathological events have been recently confirmed through *in vivo* studies, while other possible mechanisms observed in the context of SARS and MERS infections remain to be verified.

### COVID-19 and Cytokine/Chemokine Activation

In studies published during the pandemic outbreak, various cytokines and chemokines were measured to understand the full cytokine profile of severe patients at the acute phase in an attempt to elucidate the pathogenic mechanisms that led to worsening outcomes. Significant elevation of cytokines such as IL-1β, IFN-γ, IP-10, MCP-1, MCP-3, and IL-1ra were observed in critically ill patients. Additionally, Th-1 cell (IL-2, TNF-α, IL-1β, and IFN-γ) and Th-2 cell (IL-4, IL-10) -related cytokines were detectable simultaneously (Table 2) (49). Levels of lymphocytes and T cell-related CD molecules were found to be negatively correlated with cytokine changes, suggesting a potential association between cytokine storm and adaptive immunity. During the convalescent phase in mild patients, lymphocyte levels gradually returned to a normal range where the cytokines first faded and were then later undetectable. During the acute phases, the elevation in lymphocytes was not accompanied by a significant elevation in cytokines (48). This may be due to the initiation of a cellular immune response that accelerated viral clearance at the early phases, thus inhibiting cytokines' production by innate immune activation and alleviating disease severity.

The persistent secretion of IL-6 and GM-CSF that has been observed in COVID-19 patients supports the pathogenic role of atypical innate immune cells, thus suggesting their participation in COVID-19 pathogenesis (Figure 1). However, such significant elevation was not detectable in other severe cohorts (108, 109), and this led to the investigation of other possible mechanisms. In one study, IFN-α was the only cytokine that was determined to be significantly elevated. While the IFN-I inducing pathway and ISG were both activated, IFN-I gene expression was not detectable in PCMBs, suggesting that pDCs act as the main source of IFN-I (108). Additionally, IFN-I was demonstrated to be positively correlated with disease severity, and this was quite different compared to SARS infection, as abolished IFN-I signalling was previously shown to reverse lethal SARS. Taken together, these findings suggest that IFN-I might orchestrate a dysregulated immune response that leads to COVID-19 aggravation (Figure 1).

It is evident that hyperactivation of cytokines during the acute phase can result in dysregulated systemic inflammation and disease deterioration. This idea is supported by the observed elevation in D-dimers, C-reactive protein (CRP), ferritin, and procalcitonin in severe COVID-19 patients (110). Cytokine storm exerts a pathogenic rather than protective impact on the host. Careful observation revealed that the excessive cytokines and chemokines activated by macrophages (i.e., IL-6, IL-7, TNF-α, CCL-2/MCP-1, CCL-3/MIP-1α) were similar to results previously found in hemophagocytic lymphohistocytosis (HLH) and macrophage activation syndrome (MAS). Both of these conditions were unique forms of cytokine releasing syndrome (CRS) that were found in different clinical scenarios. These were treated with approved therapeutic indications of tocilizumab (IL-6 receptor inhibitor) (103). Additionally, tocilizumab (IL-6 receptor inhibitor) has exhibited therapeutic benefits in COVID-19 preliminary trials, and this is one of the most promising drug candidates to date.

### COVID-19 and Cellular Immunity

Evidence of dynamic changes in adaptive immunity between mild and severe patients was collected through longitudinal analyses of lymphocyte and subset counts. In early infection, a decrease in lymphocyte counts was observed in the severe group, and this was characterised by a dramatic loss of NK cells, B cells, CD3+, CD4+, and CD8+ T cells. During early infection, mild cases experienced a moderate increase in lymphocytes after spontaneous activation. Lymphocytes in the severe group later reached a comparable level to that of the mild group. Additionally, for the mild group, only slight differences in lymphocytes were observed at different time points (48). Another study observed an elevation in S-RBD-specific T cells and NP-specific T cells in mild patients. A strong correlation was confirmed between neutralising antibody titres and the number of virus-specific T cells (111). Similar T cell signatures were also observed from the BALF of mild patients, and this was characterised by highly expanded clonal CD8+ T cells and memory T cells. Together with the minimal level of inflammatory monocytes observed, these findings suggest a protective role for the robust adaptive response that occurred within the alveoli in mild COVID-19 patients (102). Additionally, Th1- and Th2-related cytokines were both activated and detectable during COVID-19 courses, suggesting that an extensive upregulation of adaptive immunity occurred during COVID-19 infection (49). In one clinical case of mild COVID-19, antibody producing cells and T cell immune responses (CD4 + and CD8 + T cells) reached peak levels prior to resolution of symptoms and were concurrently maintained at a steady level during the convalescence phase. Additionally, viral replication was no longer detectable after the initiation of the above responses, suggesting an effective viral clearance (112). This result again demonstrated the viability of initiating a protective adaptive immune response during early infection. Levels of the adaptive immune response could be utilised as predictive prognosis parametres and may correlate with improved clinical outcomes.

According to a pathological report of one severe COVID-19 patient, peripheral blood and lung biopsy pathology were analysed. High levels of CD4 + T cell-derived Th-17 were detected from blood through the use of flow cytometric analysis, and high levels of cytotoxic CD8 + T cells were found to be dominant in lung tissues. Excessive CD8 + T cell and Th-17 type responses were speculated to be partially responsible for the local severe lung injuries and COVID-19 exacerbation (101). In contrast, Th1 cell responses and CD8 + T cell-mediated adaptive immunity exhibited protective effects during early MERS, while the Th2 cell response (predominantly CD4 + T cell activation) is associated with a poor prognosis (51). Strong Th-17-mediated cytokine storm and the activation of related pathways were also both detected in SARS and MERS infections and played essential roles in driving pulmonary inflammatory injuries. Based on this, specific inhibitors targeting Th-17 pathway signals may provide therapeutic benefits. One recent study examined this signalling pathway and proposed that a JAK2 inhibitor (Fedratinib) could provide an effective drug option. The JAK2 signal is required for the activation of a transcription factor (i.e., STAT3) that is involved in Th17 cell differentiation. Such an inhibitor may exert a promising effect on patients with a Th-17-dominant immune profile (113).

It is clear that the adaptive immune response contributes to a certain extent to host pathogenesis. However, it is the change in breadth of adaptive immunity caused by the imbalanced Th1/Th2 activation and altered T lymphocyte function, and not the magnitude of the adaptive immune response, that results in a detrimental effect on the host. This study suggested excessive exhaustion of functioning CD8+T cells and reduced functional diversity of T cells could be predictors of disease severity in the context of COVID-19. The magnitude of CD4 + and CD8 + T cell cytokines was significantly diminished in COVID-19 patients when compared to levels in healthy controls, and markers and effective molecules related to T cell activation and regulation were either markedly decreased or increased, suggesting a subverted T cell homeostasis. In severe patients, reduction in multi-function CD4 + T cells and excessive exhaustion of CD8 + T cells leads to the progressive decline of T cell subsets, ultimately resulting in the inability to initiate an effective cellular immune response (Figure 1) (109).

### COVID-19 and Humoural Immunity

In regard to the long term effect of antibodies in COVID-19 recovered patients, one study observed elevated antibody titres as early as 1 week after the onset of symptoms, and the majority of patients experienced seroconversion within 3 weeks. Rapid increases in antibody titres have also been observed in severe cases (7), further calling into question the protective function of antibodies. In one study, antibody titres in mild patients were not consistent with viral load changes (112), and a study of immune profiles revealed the early emergence and rapid increase of activated CD8+ T cells prior to symptoms resolution. These CD8+ T cells possess the potential for increased cytotoxicity in terms of their granzyme and perforin levels, suggesting a possible role in viral clearance that further calls into doubt the protective effect from antibodies. Recent studies have also provided implications regarding the role of antibodies. Titres of antibodies are higher in elderly COVID-19 patients, and this is positively correlated to plasma CRP levels but negatively correlated to lymphocyte counts (114) and suggests a possible link between humoural and cellular immunity. Another study suggested that high antibody titres act as a risk factor of critical illness, likely due to an antibody-dependent enhancement (ADE) effect (115) (Figure 1). Coincidently, similar changes in early antibody titres were also observed in SARS studies (3). ADEs have been confirmed as an underlying pathogenesis in MERS exacerbation and were previously proposed to exert distinctive effects in SARS infection (75, 76). The protective effect of COVID-19 antibodies remains to be confirmed by further investigation of the cellular response and antibody effect. Research in regard to vaccine development requires more evidence from immunological studies of COVID-19 (116).

## Immunotherapy And Prospects

Based on global research efforts in defining the full profile of human coronavirus infection, it is now rational to speculate that SARS-CoV-2 infection does not simply lead to lethal pneumonia but also exerts a sophisticated immunopathological impact. During the SARS outbreak, interferon, ribavirin, and corticosteroid treatments improved recovery rates and reduced the length of the disease course. These treatments were initially utilised based on their broad antiviral and anti-inflammatory effects that were confirmed by *in vitro* studies. However, these treatments failed to exhibit consistent efficacy in randomised trials and could increase patient risk for hazardous short- or long-term drug-related adverse events. To date, there are still no clear benefits of any specific drugs or regimens in the treatment of SARS or MERS.

### Current Research Progress on Therapeutic Drugs

Many months have passed since the global outbreak of COVID-19. Thanks to the preliminary SARS and MERS research and the rising concerns regarding pandemic diseases, remarkable and timely research progress has been achieved. Since the COVID-19 outbreak, hundreds of drugs have been registered for clinical trials (117). Anti-viral drugs remain the first-line medication and can be classified into repurposed, investigational, or novel drugs (118). Repurposed drugs (Ribavirin, Lopinavir/Ritonavir, and Arbidol) previously proven effective in influenza or HIV infection were suggested and required further verification by clinical trials. Investigational drugs such as Remdesivir (nucleotide analogue prodrug) have recently drawn attention due to the inconsistent efficacy observed from randomised multi-centre trials and case-control reports. Favipiravir (broad-spectrum antiviral agent) is another investigational drug that has shown limited benefits that were confined to mild patients. Novel drug design requires more supporting evidence from laboratory research. In short, antiviral drugs that take years of clinical trials to prove their efficacy are encountering setbacks and show little progress at the early phases. Several treatment approaches that had first proven effective *in vitro* later failed to exhibit therapeutic benefits in clinical trials, and these are the same predicaments that were encountered during the SARS and MERS epidemics. These setbacks inspire us to explore the potential for alternative treatment approaches that could show similar benefits and could be promptly used during this ongoing pandemic.

Due to the complex pathogenesis and broad pathological effect on host immunity exerted by COVID-19, antiviral drugs alone are ineffective in improving clinical outcome. Adjunctive anti-inflammatory therapies that target critical conditions, such as ARDs and cytokine release syndrome (CRS), may slow disease progression and minimise the need for advanced supportive care. Although dynamic immunological changes were detected among COVID-19 patients, there is no clear evidence for clinical improvement, and these treatments may actually worsen clinical outcomes. In regard to previous research work on SARS and MERS, it is evident that immunopathology is involved in and drives host systemic damage, further highlighting the urgent need for an immunotherapeutic approach.

### Implication of Biological Agents

Here, we focus mainly on biological agents that possess anti-inflammatory effects. IL-6 receptor inhibitors possess a rational immunotherapeutic basis, as they target the initiating upstream cytokine in the inflammatory pathway to prevent cascade activation at the very first step. IL-6 could interplay with various host cell pathways involved in acute phase reactions, innate and adaptive immunity activation, coagulation, complements, endothelial cells, and haematopoiesis to exert system-wide impacts while inducing systemic inflammation.

IL-6-derived cytokine storm is known to be induced in response to viral infection, autoimmune disease, and inflammatory disease (119). The most prevalently used IL-6 inhibitor, Tocilizumab (IL-6R monoclonal antibody), was approved to treat sJIA (Juvenile idiopathic arthritis) and chimeric antigen receptor (CAR) -T cell therapy-derived CRS. Both of these conditions are characterised by an overwhelming release of cytokines that are involved in macrophage activation syndrome (MAS). Recently, Tocilizumab was incorporated as a treatment option in COVID-19 management guidelines to be used in severe or high-risk conditions with elevated concentrations of IL-6. Several studies have observed MAS-like cytokine profiles in COVID-19 patients and have demonstrated its association with disease severity, thus providing sufficient evidence for IL-6R blockade treatment. Recently, one preliminary study showed rapid improvements in respiratory function and oxygen requirement after tocilizumab treatment (120). A multi-centre clinical trial that recruited more than 500 patients is now under way (121), and the results of this trial will be soon provide convincing research outcomes. Another therapeutic basis for Tocilizumab is that it demonstrates anti-inflammatory effects without diminishing the cytotoxic activity by activated T cells, thus allowing for effective viral clearance. Moreover, timing and indications of IL-6 blockade therapy should be based on the disease severity and patient IL-6 levels, as early or excessive IL-6 blockage may delay the immune response and viral clearance.

Other biological agents that possess immunomodulatory or anti-inflammatory effects have also been suggested to have potential therapeutic benefits in COVID-19 management. These molecules target an important immune-checkpoint or an upstream cytokine. Monoclonal antibodies or inhibitors targeting IL-1 and IL-17 could inhibit pro-inflammatory molecules and could influence the activation of innate and adaptive immunity (113, 122). Inhibitors targeting PD-1 and TIGIT have proven effective in the treatment of HCV chronic infection by preventing lymphocyte exhaustion and restoring anti-viral immunity (123). Similar impairment of lymphocyte function was also observed in the context of COVID-19 and was demonstrated to be related to disease severity (124); however, the participating role of PD-1 in COVID-19 infection, particularly in regard to lymphocyte activation, should be further discussed. Another signalling molecule, NKG2A, was recently found to be activated in COVID-19 lymphocytes and was previously demonstrated to be involved in lymphocyte functional exhaustion (125). NKG2A+ inhibition that was achieved with certain anti-viral drugs later helped to restore cytotoxic lymphocyte counts and prevent T cell function exhaustion in COVID-19, indicating certain therapeutic benefits. NKG2A may influence an important immune checkpoint that is correlated with disease control in COVID-19 patients (100).

### Challenges and Therapeutic Implications of Antibody-Mediated Responses

The successive emergence of highly contagious viral infections, such as MERS-CoV, Zika, Ebola, and SARS-CoV-2, has resulted in unprecedented burdens to the global community. The recent occurrence of a bat-derived coronavirus disease has led to an ongoing pandemic and has emerged as a major global issue that must be resolved. The current global responses have reflected the lack of preparation about confronting the emergence of a novel viral infection. There is an urgent need to clarify the underlying pathogenesis and to fully understand the human-virus interaction to allow for the development of appropriate interventions that will allow us to control this pandemic. Among the substantial number of research achievements, novel interpretation of previously confirmed discoveries has provided valuable insights into COVID-19 therapeutic strategies. In regard to serum antibody function, it was confirmed that the presence of pre-existing dengue virus (DENV) antibodies could enhance viral entry during Zika virus exposure, likely due to the sero-cross reactivity between the two viruses (126). This ADE effect was believed to have contributed to the enhanced pathogenesis of the Zika epidemic found in Latin America, where DENV infection is also prevalent (127). This hypothesis was confirmed by *in vivo* studies and is consistent with clinical results (128). Similarly, sero-cross reactivity was also observed for SARS-CoV and MERS-CoV, and this was elicited by a conserved N protein epitope that is commonly found in coronaviruses (80). In another study, initial infection with SARS-CoV could lead to a significant decrease in MERS-CoV titres. The presence of a cross-reactive T cell response was identified to accelerate MERS-CoV clearance (129). Given that SARS-CoV-2 shares similar viral protein structures with other coronaviruses, it is reasonable to hypothesise that sero-cross reactivity, such as ADE, may occur during COVID-19 infection. It is of great significance to elaborate on such topics and to confirm the underlying implication of sero-cross reactivity in COVID-19 pathogenesis.

Vaccination is established as the best strategy to produce herd immunity for the control of pandemics, and vaccines have eradicated a number of infectious diseases such as smallpox, poliomyelitis, and measles. To ensure vaccine safety, it is important to avoid eliciting humoral immune responses, to reduce titres of neutralising antibodies (nAb), and to avoid the lasting of neutralising effects of non-neutralising antibodies during vaccine development.

Among these, antibody dependent enhancement (ADE) was identified as a novel antibody-mediated response that raised concerns during SARS vaccine development. The molecular basis of ADE mechanisms has been discussed in each section and has been demonstrated to be related to enhanced viral replication in *in vitro* studies. In clinical scenarios, primary infection or vaccination could lead to the production of antibodies that are sub-neutralising or non-neutralising for secondary infection and these antibodies were suspected to potentiate clinical worsening through the ADE effect. In fact, ADE does not always elicit a pathological effect and instead may have other implications during coronavirus infection. In ADE-observed SARS-vaccine-immunised animals, these antibodies were still able to mediate protective neutralising effects without aggravation of lung pathology (72). Another study demonstrated an enhanced infection of macrophages via an ADE mechanism without the observation of productive replication, and this closely resembled the abortive infection previously observed in SARS. It was also stated that sufficient viral entry via the ADE mechanism not only depends on the presence of FcγRs but also requires the activation of cytoplasmic signalling pathways. This explains the discrepancies in ADE enhancing abilities found between different types of FcγRs expressing cells (130). MERS studies have also demonstrated the association between antibody titres and viral entry into cells that express viral receptors, Fc receptors, and both receptors. It was stated that determinants of ADE-enabling Mab dosages might include binding affinities or expression levels of receptors found in special tissues (76). All of these results revealed the complex functions of antibodies and may guide vaccine design and provide novel conception for vaccine strategies. As demonstrated in an *in vitro* study, the precise role of antibodies may differ under different clinical situations. ADE may only occur during certain clinical phases with optimal titres of antibodies and immune cells that are ready for viral entry. Moreover, such effects may only be observed in a small subset of patients whose immune responses were initiated in a manner favourable to ADE.

*In vitro* results did provide a fundamental molecular basis for novel mechanisms and raised concerns on the potential pathogenesis of ADE. However, results observed *in vitro* fail to account for the dynamic activation of both innate and adaptive immunity and do not provide a full demonstration of effector mechanisms (such as complement activation and phagocytosis). Without extensive demonstration of ADE in coronavirus-infected animal models, it is likely too early to attribute ADE to COVID-19 pathology (131). Improper attribution of ADE to COVID-19 pathogenesis may slow the progress of vaccine development. Concurrently, with better understanding of ADE signalling and antibody function, these experiments provide numerous theoretical principles for vaccine development and point to potential approaches for reducing the likelihood of ADE, such as by developing a spike-protein based subunit vaccine that is lacking RBD.

The coronavirus S protein was capable of inducing both neutralising antibodies and protective T cell responses after infection. Characterised with a high immunogenicity and various participating roles in receptor binding and membrane fusion during viral entry, the S protein could serve as a target antigenic component in vaccine development (132). Despite its neutralising protection, anti-spike protein antibodies were identified to be the most prevalent antibodies involved in ADE effects in both SARS and MERS infections. In view of this, scientists have recently proposed the concept of developing a multiple-epitope peptide vaccine that aims to concurrently initiate B cell, CD4+ T cell, and CD8+ T cell responses that can drive both humoral and cellular immunity after immunisation. Based on this, the impacts of ADE could be minimised through the clearing of infected antigen-presenting cells by promptly activated CD8+ T cells (133). This may serve as a valuable vaccine option for animal experiments and could benefit the progression of future vaccine studies of SARS-CoV-2.

## Conclusion

Amid the ongoing SARS-CoV-2 pandemic, it is rational to begin with a comparative literature review between the three highly related human coronaviruses that have triggered global pandemics in the twenty first century. To clarify the underlying pathogenesis of these viruses to allow for the development of effective therapeutic strategies, it is necessary to study the contributing role of immunological changes and host interactions among these three types of infections to point out similarities and differences for future investigation. Moreover, novel insights and research strategies related to the COVID-19 pandemic could be gained from previous confirmed findings in coronavirus studies. Based on this, we have extensively assessed both clinical and experimental studies and gathered the important findings in this review.

We first discussed the evolutionary history and virology of lethal human coronaviruses. Next, we provided extensive information for each of these three coronavirus infections. In each coronavirus section, we first introduced clinical and immunological features observed in clinical settings, and these included epidemiology, clinical manifestation, prognosis, and remarkable immunological features. Then we elaborated on the distinct immunological changes within clinical courses. We deliberately divided clinical courses into phases according to the host immunological manifestations and disease severity to better illustrate the involved pathogenesis mechanisms in accordance with clinical situations. Distinct immunological features and their potential implications were mentioned, respectively, in each coronavirus section. In short, these three coronavirus infections shared similar immunological features that exert pathological impacts and could be classified into four main aspects: (1) viral replication in innate immune cells, (2) dysregulated immune response, (3) cytokine storm, and (4) antibody-mediated response. Similarities and differences were compared in each of the coronavirus sections. In the MERS-CoV section, the immunological features were mostly compared to those of SARS-CoV due to previous study designs as well as cross-similarities between these two infections. For SARS-CoV-2, aside from basic knowledge related to clinical infection, the potential immunopathogenesis was hypothesised based on supporting evidence from recent findings. These were classified according to the type of immune response involved. Most of these recently evolved hypotheses largely depend on confirmed SARS-CoV and MERS-CoV pathogenesis and COVID-19 clinical studies, while some of these have already been confirmed in *in vitro* studies. For the last section, we discussed the therapeutic strategies and implication of immunotherapy in the context of COVID-19. Potential therapeutic and preventive approaches were introduced with respect to their theoretical basis. We focused on therapeutic approaches and important issues related to the regulation of the immune system. Approaches such as biological agents, ADE effect, and vaccination were primarily discussed.

This review aimed to provide research insights and supporting evidence for understanding the pathogenesis and therapeutic strategies of COVID-19, particularly via an immunological prospective. Most of the mentioned underlying pathogenesis of coronavirus infections were preliminary and could only be observed in *in vitro* studies. These provided a strong molecular basis and could guide future demonstrations in animal studies. Some of these mechanisms have been confirmed to be strongly correlated to clinical prognosis and disease outcome, and thus underlined their potential contributing roles in pathogenesis and clinical manifestations. In review of most of the existing studies, growing numbers of research findings regarding coronavirus and other viral infections have made much progress, and these could facilitate future clinical studies and large trials to verify the laboratory-confirmed hypotheses.

## Author Contributions

YZ analyzed the data and wrote the paper. BN and BL designed and revised this paper. All authors contributed to the article and approved the submitted version.

## Conflict of Interest

The authors declare that the research was conducted in the absence of any commercial or financial relationships that could be construed as a potential conflict of interest.
